# Complete Surgical Resection Following Neoadjuvant Dabrafenib Plus Trametinib in *BRAF^V600E^*-Mutated Anaplastic Thyroid Carcinoma

**DOI:** 10.1089/thy.2019.0133

**Published:** 2019-08-19

**Authors:** Jennifer R. Wang, Mark E. Zafereo, Ramona Dadu, Renata Ferrarotto, Naifa L. Busaidy, Charles Lu, Salmaan Ahmed, Maria K. Gule-Monroe, Michelle D. Williams, Erich M. Sturgis, Ryan P. Goepfert, Neil D. Gross, Stephen Y. Lai, Gary Brandon Gunn, Jack Phan, David I. Rosenthal, Clifton David Fuller, William H. Morrison, Priyanka Iyer, Maria E. Cabanillas

**Affiliations:** ^1^ Division of Surgery, Department of Head and Neck Surgery, The University of Texas MD Anderson Cancer Center, Houston, Texas.; ^2^ Division of Internal Medicine, Department of Endocrine Neoplasia & Hormonal Disorders, The University of Texas MD Anderson Cancer Center, Houston, Texas.; ^3^ Department of Thoracic/Head & Neck Medical Oncology, The University of Texas MD Anderson Cancer Center, Houston, Texas.; ^4^ Department of Diagnostic Imaging, The University of Texas MD Anderson Cancer Center, Houston, Texas.; ^5^ Department of Pathology, The University of Texas MD Anderson Cancer Center, Houston, Texas.; ^6^ Department of Radiation Oncology, The University of Texas MD Anderson Cancer Center, Houston, Texas.; ^7^ Diabetes, Endocrinology & Metabolism, PeaceHealth Medical Group-Oregon, Eugene, Oregon.

**Keywords:** dabrafenib, trametinib, pembrolizumab, squamous, sarcomatoid, anaplastic thyroid cancer, dedifferentiated, undifferentiated, targeted therapy, chemotherapy, surgery

## Abstract

***Background:*** When achieved, complete surgical resection improves outcomes in anaplastic thyroid carcinoma (ATC). However, most ATC patients present with advanced inoperable disease, often with impending airway obstruction, increased hemorrhage risk, and significant dysphagia. Novel treatment strategies are critically needed to improve disease control and decrease locoregional morbidity. The objective of this study was to determine the feasibility and effectiveness of a neoadjuvant regimen by using dabrafenib with trametinib followed by surgical resection in patients with initially unresectable *BRAF^V600E^*-mutated ATC.

***Methods:*** Case series of six consecutive patients with *BRAF^V600E^*-mutated ATC diagnosed between January 2017 and February 2018. Pathologic confirmation of ATC was obtained before treatment. *BRAF^V600E^* status was ascertained via immunohistochemistry or sequencing of circulating tumor DNA. All patients received dabrafenib and trametinib (DT) followed by surgical resection and adjuvant chemoradiation. Three patients also received pembrolizumab.

***Results:*** Complete surgical resection was achieved in all patients. Histopathologic analyses of resected specimens showed high pathologic response rates with significantly decreased ATC viability and residual papillary thyroid carcinoma components. Overall survival at six months and one year was 100% and 83%, respectively. Locoregional control rate was 100%. Two patients died of distant metastases without evidence of locoregional disease at 8 and 14 months from diagnosis. The remaining four patients had no evidence of disease at the last follow-up.

***Conclusions:*** We report the first series in the literature of *BRAF^V600E^*-mutated ATC patients with locoregionally advanced disease treated with DT followed by surgical resection. We demonstrated feasibility of complete resection, decreased need for tracheostomy, high pathologic response rates, and durable locoregional control with symptom amelioration.

## Introduction

Anaplastic thyroid carcinoma (ATC) is one of the most lethal human malignancies with a median overall survival of five months (1). When achievable, complete surgical resection has been shown to improve outcomes in ATC (2–4). However, the vast majority of patients present with inoperable disease due to involvement of critical structures such as the carotid artery, larynx, trachea, esophagus, and/or mediastinum.

The *BRAF^V600E^* mutation has been detected in up to 25–45% of ATCs (5–8). The combination of dabrafenib and trametinib (DT) became the first targeted regimen approved for ATC based on a clinical trial that showed high response rates and significantly improved survival (9,10). However, the trial enrolled patients with good performance status only and excluded many patients with advanced disease (11). Herein, we report our experience with neoadjuvant DT followed by complete surgical resection in six *BRAF^V600E^*-mutated ATC patients who initially presented with unresectable disease.

## Materials and Methods

Patients included in this series were consecutive *BRAF^V600E^*-mutated ATC patients treated at our institution who presented between January 2017 and February 2018 with unresectable disease and who did not participate in a clinical trial. Within the study period, five additional *BRAF^V600E^-*mutated ATC patients were treated at our institution outside of a clinical trial and not included in this series. One patient presented with resectable disease and underwent primary surgery followed by adjuvant chemoradiation. DT were given for locoregional and distant progression 15 months after completion of adjuvant chemoradiation. Four patients received DT but did not undergo subsequent surgical resection, including one patient who declined surgery despite eligibility. Two of the four patients presented with large-volume distant metastases that continued to progress on DT. The remaining patient had progressive locoregional as well as distant disease on DT.

**FIG. 1.**
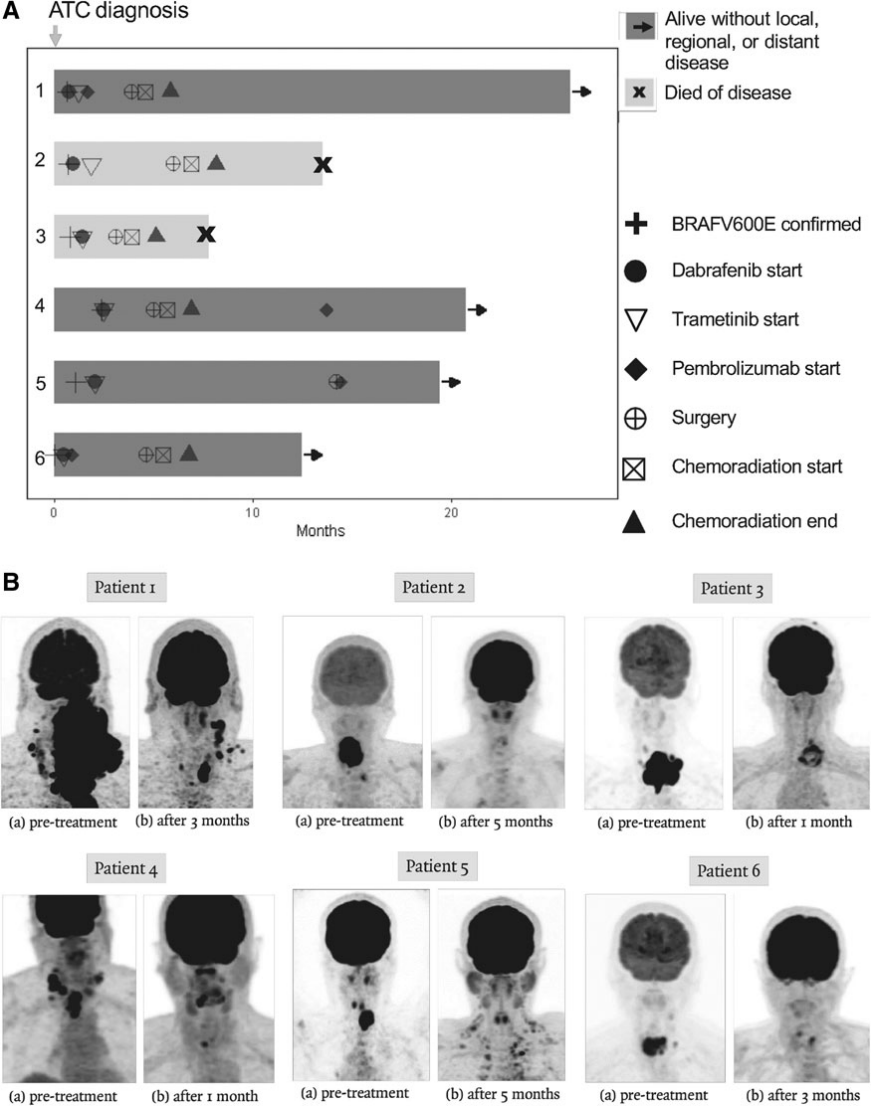
Summary of treatment course and representative imaging. Swimmer's plot of patients' treatment course (**A**); representative PET/CT images before and after neoadjuvant treatment, before surgical resection (**B**). PET/CT, positron emission tomography–computed tomography.

Pathologic confirmation of ATC was obtained in all cases before treatment initiation. Diagnoses of ATC were made from core biopsies obtained at our institution before the initiation of DT by a head and neck pathologist with expertise in ATC based on histopathologic evaluation and immunohistochemistry studies. *BRAF^V600E^* status was ascertained by immunohistochemistry or sequencing of circulating tumor DNA (cfDNA). Pretreatment tumor-based somatic mutation testing was performed within our CLIA-certified laboratory by using next-generation sequencing targeted mutation panels on surgical or core biopsy samples in five out of six patients. In patient 1, pretreatment mutation testing was obtained via cfDNA analysis due to insufficient biopsy material to perform tumor-based mutation testing. In addition to the histopathologic features and immunohistochemistry consistent with ATC, all six patients presented with a clinical picture consistent with ATC, namely, a very rapidly growing thyroid cancer with significant invasion of neck structures. The preoperative imaging for these six patients in Figure 1 demonstrates the extent of locoregionally advanced disease. Pretreatment staging included computed tomography (CT) neck, chest, magnetic resonance imaging brain, and whole-body fluorodeoxyglucose positron emission tomography–computed tomography (PET/CT). Cross-sectional imaging was repeated on restaging to determine surgical resectability.

When DT were not immediately accessible, cytotoxic chemotherapy (paclitaxel ± carboplatin) was utilized as bridging chemotherapy. DT were given orally at doses of 150 mg twice daily and 2 mg daily, respectively. In patients unable to swallow pills, dabrafenib capsules were dissolved into a suspension and trametinib tablets were crushed. The medications were administered in the modified oral form or via the gastrostomy tube until patients were able to swallow pills. Three out of six patients also received pembrolizumab before or after surgical resection. Trametinib was held five to seven days before surgery, and dabrafenib was held the previous day or the day of surgery. Surgical resection was followed by adjuvant chemoradiation. Except for 1 patient who declined, a total of 60 Gy in 30 fractions was administered concurrent with chemotherapy (paclitaxel and carboplatin, or cisplatin alone). Patient characteristics and treatment courses are shown in Table 1 and Figure 1, respectively. A summary of surgical procedures, surgical pathology, and pretreatment mutation status is shown in Table 2.

**Table 1.** Demographic and Clinical Characteristics of the Patients

*Characteristic*	N	*%*
Age at diagnosis, years		
Median: 59		
*SD*: 9.9		
Range: 46–73		
Sex		
Male	2	33
Female	4	67
Method of *BRAF^V600E^* detection		
Immunohistochemistry	3	50
cfDNA	3	50
T stage at diagnosis		
T4a	0	0
T4b	6	100
N stage at diagnosis		
N0	0	0
N1a	1	17
N1b	5	83
M stage at diagnosis		
M0	4	67
M1	2	33
Bridging chemotherapy		
Carboplatin and abraxane	1	17
Abraxane only	1	17
Paclitaxel	2	33
None	2	33
Surgical resection		
R0	4	60
R1	2	40
Dabrafenib/Trametinib administration		
Via gastrostomy tube	2	33
Modified oral administration	2	33
Standard oral administration	2	33
Post-op complications		
Wound infection	1	17
Temporary unilateral vocal cord paresis	1	17
Pulmonary embolism	1	17
Adjuvant chemoradiation		
Yes	5	83
No	1	17
Duration of neoadjuvant treatment, months		
Median: 3.6		
*SD*: 3.5		
Range: 1.6–12		
Vital status		
Alive without evidence of disease	4	67
Died of disease	2	33
Duration of follow-up from diagnosis, months		
Median: 16.5		
*SD*: 6.6		
Range: 7.8–26.0		
Duration of follow-up from start of BRAF-Directed therapy, months		
Median: 15.0		
*SD*:6.4		
Range: 6.4–25.2		

cfDNA, circulating cell-free DNA; *SD*, standard deviation.

**Table 2.** Summary of Surgical Procedures, Anaplastic Thyroid Carcinoma Viability, Resected Tumor Histopathology, and Pretreatment Mutation Profile

	*Patient 1*	*Patient 2*	*Patient 3*	*Patient 4*	*Patient 5*	*Patient 6*
Procedures	TT, central ND, bilateral ND, limited resection of esophageal muscularis	TT, central ND, right ND, *en bloc* resection of right RLN	TT, central ND, left ND, *en bloc* resection of left RLN, shave resection of trachea	TT, central ND, revision right ND, left ND, resection of dermal metastasis	TT, central ND, left level IV ND, and resection of occipital scalp lesion	TT, central ND
Resection of ATC	Complete	Complete	Complete	Complete	Complete	Complete
Residual (R) tumor classification	R1	R0	R1	R0	R0	R0
Resected ATC viability, %^a^	50	5	<5	0	0	0
Additional component in resected tumor	PTC	PTC	PTC	PDTC, PTC	PTC	PTC
Pretreatment tumor mutations	*BRAF^V600E^, TP53^R175H^, EGFR G322S, BRAF amplification*^b^	*BRAF^V600E^, TP53^Q331 a^*	*BRAF^V600E^, TP53^D208V^, CDKN2A^pE88 a^*	*BRAF^V600E^*	*BRAF^V600E^, ATM^I1986V^, MCL1 duplication*	*BRAF^V600E^*

^a^ Indicated viability of the ATC component of the postneoadjuvant treatment resected primary thyroid tumor.

^b^ Detected in cell-free DNA.

ATC, anaplastic thyroid carcinoma; ND, neck dissection; PDTC, poorly-differentiated thyroid carcinoma; PTC, papillary thyroid carcinoma; RLN, recurrent laryngeal nerve; TT, total thyroidectomy.

## Results

The first *BRAF^V600E^*-mutated ATC patient treated at our institution with a neoadjuvant approach was reported separately (11). In brief, this 60-year-old male presented with T4bN1bM0 ATC surrounding the larynx, esophagus, and carotid arteries and was treated with DT. Pembrolizumab was added before surgical resection due to disease progression. R1 resection was performed complicated by a wound infection requiring incision and drainage. He underwent adjuvant chemoradiation plus pembrolizumab. DT were not initially resumed after chemoradiation and the patient developed a local recurrence. With subsequent resumption of DT, the local recurrence receded. At 26 months after diagnosis, this patient is alive and remains free of disease on DT and pembrolizumab.

**FIG. 2.**
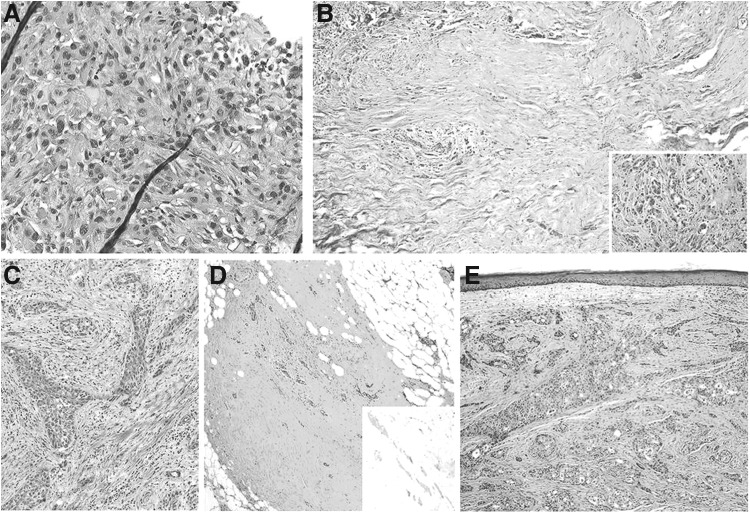
Representative histopathology of pre- and post-BRAF/MEK-treated anaplastic thyroid carcinomas. Residual tumor viability and representative histopathology. (**A, B**) Show representative histologic slides from Patient 2. (**C–E**) Show representative slides from Patient 5. (**A**) Pretreatment biopsy showing pleomorphic cells with eosinophilic cytoplasm with squamoid features growing in cords. The background is desmoplastic with scattered inflammatory infiltrate (hematoxylin and eosin stain, 400 × magnification). (**B**) Post-treatment surgical resection at low power magnification (hematoxylin and eosin stain, 40 × magnification) shows large fibrotic areas of the treated tumor bed with scattered viable tumor nests (left side); the inset shows a viable tumor area with desmoplastic stroma (100 × magnification). (**C**) Diagnostic core biopsy shows irregular squamoid tumor cells in a fibrotic stroma (hematoxylin and eosin stain, 100 × magnification). (**D**) Post-treatment, the surgical resection shows large areas of fibrosis with scattered follicles of residual well-differentiated papillary thyroid carcinoma that retain BRAF^V600E^ expression by immunohistochemistry (inset) (100 × magnification); (**E**) Post-treatment area that showed progression in the scalp shows viable anaplastic thyroid carcinoma growing in squamoid nests similar to the pretreatment biopsy (hematoxylin and eosin stain, 100 × magnification). This tumor also retains BRAF^V600E^ immmunoexpression (immunoslide not shown).

Patient 2 was a 48-year-old female with T4bN1bM0 ATC (Fig. 2A, B). Given carotid encasement and laryngoesophageal invasion, she was not a candidate for primary surgical resection. DT were started. Initial scans after one month of treatment showed >50% tumor reduction. Dysphagia and dyspnea markedly improved. Restaging scans at three months showed further response and she became eligible for surgical resection, which included total thyroidectomy, central neck dissection, and right level II–V neck dissection. Due to tumor involvement, the right recurrent laryngeal nerve was resected *en bloc*. An R0 resection was achieved. A 1.0-cm unifocal tumor with ATC and papillary thyroid carcinoma components and one level VI lymph node with a 0.5-cm focus of ATC was seen on surgical pathology. Her initial postoperative course was uncomplicated, and she was discharged home on postoperative day (POD) 4. On POD22, she developed a segmental pulmonary embolus necessitating hospitalization and anticoagulation. On POD28, she began adjuvant concurrent chemoradiation. She was restarted on DT after a significant delay of five weeks after chemoradiation completion due to patient preference. She had no evidence of disease until one year from diagnosis when she developed bone metastases. At 14 months from diagnosis, she died of progressive distant metastases but remained free of locoregional disease.

Patient 3 was a 69-year-old female with T4bN1aM0 ATC, which was unresectable due to tracheal involvement and extensive disease surrounding the carotid. Within one month of starting DT, her dyspnea resolved and she resumed a full oral diet. Restaging evaluation showed significant reduction of the primary tumor and lymphadenopathy as well as separation from the carotid. As such, she was taken to the operating room for a total thyroidectomy, central and left neck dissection (levels II–IV), and shave resection of the trachea. The left recurrent laryngeal nerve was resected *en bloc* due to tumor encasement. Pathology showed a significant treatment effect with minimal ATC viability. Clusters of residual ATC were noted along with microscopic foci of papillary thyroid carcinoma. Therapy-related changes were observed in lymph nodes without viable tumor. R1 resection was achieved due to microscopic involvement of the resection margin by papillary thyroid carcinoma. She was discharged on POD4 without complications. For adjuvant treatment, although she completed radiation, the last cycle of chemotherapy was omitted due to dehydration. DT were not resumed after completion of chemoradiation. Although she remained disease free locoregionally, she developed distant metastases involving the brain, lung, adrenals, spine, and chest wall. She received one dose of whole-brain irradiation and ultimately died of distant disease nearly eight months after diagnosis.

Patient 4 was a 58-year-old male with a T4bN1bM0 ATC who developed progressive dysphagia requiring gastrostomy tube placement. Surgical resection at another institution was aborted due to encasement of the trachea and esophagus. On evaluation at our institution, CT neck revealed unresectable disease involving the trachea, esophagus, and carotid arteries and extending to the thoracic inlet. As such, DT were initiated. Within one month, he showed a dramatic response with resolution of dysphagia, dyspnea, and resumption of an oral diet. However, a 3-mm nodule was noted in his previous surgical incision that was suggestive of dermal metastasis. He underwent a total thyroidectomy, central neck dissection, revision right level III–IV neck dissection, and left level II–V neck dissection. R0 resection was achieved. Bilateral recurrent laryngeal nerves were preserved. Pathology showed multifocal residual clusters of poorly differentiated thyroid carcinoma and microscopic papillary thyroid carcinoma with a marked therapy effect. A 0.3-cm focus of poorly differentiated thyroid carcinoma was noted within the previous scar. One lymph node showed a 1.5-mm focus of metastatic thyroid carcinoma. He had an uncomplicated postoperative course and was discharged home on POD2. Eight days after completion of chemoradiation, he resumed DT. Pembrolizumab 200 mg every 3 weeks was also added. At more than 20 months from diagnosis, he remained on DT and pembrolizumab and had no evidence of local, regional, or distant disease.

Patient 5 was a 73-year-old female with T4bN1bM1 ATC (Fig. 2C–E). The patient developed bilateral vocal cord paralysis and underwent a tracheostomy before presentation at our institution. Initial staging showed tracheoesophageal invasion as well as metastatic disease involving the lungs, liver, rib, and occipital scalp. She was treated with DT, achieving near-complete metabolic response on PET/CT. However, after 12 months of DT, she developed marked progression in the occipital metastasis with stable locoregional disease. An R0, total thyroidectomy, central neck dissection, left level IV neck dissection, and resection of occipital scalp lesion were performed. Given findings of bilateral vocal cord paralysis preoperatively, the tracheostomy was not removed. She had no postoperative complications and was discharged on POD2. Pathology demonstrated microscopic foci of papillary thyroid carcinoma in the thyroid and two lymph nodes (Fig. 2D). A 2.7-cm ATC was identified in the scalp metastasis (Fig. 2E). She was started on pembrolizumab (2 mg/kg IV) and DT but declined adjuvant chemoradiation. At 6 months after surgery, she had no evidence of local, regional, or distant disease and is alive at 19 months after diagnosis.

Patient 6 was a 46-year-old female with T4bN1bM1 ATC who presented with unresectable primary disease involving the left carotid, trachea, and esophagus as well as metastatic disease involving the lungs, pleura, and pericardium. The patient was started on DT concurrently with pembrolizumab 200 mg every 3 weeks. She had a dramatic response with marked reduction of primary disease and a resolution of metabolically active systemic disease. After five cycles of pembrolizumab, she underwent an R0 total thyroidectomy and central neck dissection. She had an uncomplicated postoperative course and was discharged home on POD2. Pathology showed residual papillary thyroid carcinoma in the thyroid as well as in seven lymph nodes. No residual ATC was seen. She continued pembrolizumab during chemoradiation. Two weeks after completion of chemoradiation, DT were resumed. At 12 months after diagnosis, she is free of local, regional, or distant disease.

## Discussion

Although distant metastasis are found in ∼50% of ATC patients at presentation, the immediate cause of death in the majority of patients relates to local complications such as airway obstruction, catastrophic hemorrhage, or circulatory failure due to compression of mediastinal vasculature (12–14). Patients are often faced with the difficult decision of a permanent tracheostomy with a heightened risk of major hemorrhage versus suffocating from their locoregional disease. Locally advanced disease also leads to severe pain and dysphagia, necessitating feeding tube placement. In a limited subset of patients with resectable disease at presentation, multimodality treatment, including surgical resection, has been associated with improved survival (3,4,14–16). However, for the majority of patients who present with advanced T4b disease, radical resection, including total laryngectomy and/or esophagectomy, is associated with increased morbidity and is not advocated in view of rapid recurrence, distant disease, and poor survival outcomes.

Neoadjuvant utilization of DT provides an approach to achieve locoregional control and symptom management without radical surgery. The approach has been used in *BRAF*-mutated melanoma and was found to be superior to surgery alone (17). In this series, complete resection was achieved in all patients without tracheostomy or radical resection, including in the re-operative setting. One patient in this series presented to our institution with bilateral vocal cord paralysis and a previously placed tracheostomy that remained. High pathologic response rates were observed in this study. All surgically resected specimens showed significant reduction in ATC viability (Table 2). Intriguingly, well-differentiated components remained in all resected specimens, with papillary thyroid carcinoma being the most common. Findings from this series highlight the significant intratumor heterogeneity and clonal evolution of ATC.

In our experience, this approach is feasible in ATC patients without distant metastasis at presentation and in selected patients whose distant disease responds favorably to neoadjuvant treatment. *BRAF^V600E^* status can be ascertained within days from biopsy by immunohistochemistry or cfDNA sequencing (18–20). Rapid diagnosis and treatment initiation are essential to avoid tracheostomy and local complications. In this series, we utilized bridging chemotherapy in cases where DT were not readily available after initial diagnosis due to delays in obtaining the drugs or receiving insurance approval. We did not see any significant clinical disease responses related to the bridging chemotherapy. Since the FDA approval of DT, timely access to DT has significantly improved. It is our preference to initiate DT as soon as possible after confirmation of ATC diagnosis and *BRAF^V600E^* status. Similar to prior reports utilizing DT, responses to BRAF inhibitors have been observed within days (21–23). In our series, eligibility for surgical resection occurred within weeks of DT initiation.

Complications including venous thromboembolisms and wound infections were observed in our patients and led to treatment interruption as previously reported (17). Due to the antiangiogenic properties associated with MEK inhibitors that could impair wound healing, our approach has been to stop trametinib five to seven days before surgery (24). Dabrafenib is held the day before or on the day of surgery. Both drugs are restarted as soon as the surgical wound has healed, while the patient awaits radiation. Adjuvant chemoradiation can be initiated within two to three weeks of surgery, at which time DT are held due to the risk of exaggerated acute toxicity. DT is resumed as soon as the patient recovers from radiation.

Although two out of six patients in this series went on to develop distant metastases after surgical resection and adjuvant chemoradiation, they remained free of locoregional disease. As a result, their quality of life was preserved for a longer duration despite disease progression, as they were able to maintain their ability to breathe, eat, and communicate. Our experience suggests that DT should be resumed after chemoradiation. Due to the aggressive nature of ATC and known patterns of treatment failure, we believe that patients treated with this approach remain at high risk for disease progression due to distant metastases after complete surgical resection and adjuvant chemoradiation. We hypothesize that a delay in reinitiating DT after adjuvant chemoradiation led to distant metastases in patients 2 and 3. As such, it is our current approach to minimize time without systemic therapy in these patients. DT is resumed as soon as possible after completion of adjuvant chemoradiation. Checkpoint inhibitors such as pembrolizumab may prolong disease-free survival when added to DT. All patients in whom pembrolizumab was added to DT remained alive and disease-free. These four patients are currently maintained on DT and pembrolizumab.

This case series of six patients provides initial evidence that neoadjuvant treatment strategies based on DT may improve outcomes in *BRAF^V600E^*-mutated ATC patients, whereas studies with larger sample sizes are required to evaluate the effect on progression-free survival, overall survival, and quality of life.

In conclusion, this is the first series in the literature to illustrate the feasibility and effectiveness of a neoadjuvant approach using DT in patients with locoregionally advanced *BRAF^V600E^*-mutated ATC. Further studies are required to systematically evaluate the effect of this strategy on overall survival, progression-free survival, and quality of life. Meanwhile, we suggest that physicians should pursue timely *BRAF^V600E^* testing in all patients with ATC, including those presenting with locoregionally advanced disease. DT should be given to patients with *BRAF^V600E^*-positive disease and surgical resection can be considered in suitable candidates who experience a significant response.
